# A novel mutation in the calcium-sensing receptor gene in an Irish pedigree showing familial hypocalciuric hypercalcemia: a case report

**DOI:** 10.1186/1752-1947-4-349

**Published:** 2010-10-29

**Authors:** Wael F Elamin, Olivier de Buyl

**Affiliations:** 1 Bantry General Hospital, Bantry, Co. Cork, Ireland; 2 Elrazi College of Medical Sciences and Technology, Khartoum, Sudan

## Abstract

**Introduction:**

Familial hypocalciuric hypercalcemia is a rare autosomal dominant disorder characterized by asymptomatic and non-progressive hypercalcemia due to mutations of the calcium-sensing receptor gene. Disorders of calcium metabolism are very common in the elderly, and they can coexist with familial hypocalciuric hypercalcemia in affected families.

**Case presentation:**

We describe an Irish family with hypercalcemia and hypocalciuria. The proband, an 80-year-old Irish woman, presented with hypercalcemia, relative hypocalciuria, and an elevated parathormone level. She also had chronic kidney disease stage 3 and vitamin D deficiency. Two of her sons were also found to be hypercalcemic and hypocalciuric. DNA sequencing identified a novel missense inactivating mutation in the calcium sensing-receptor gene of the proband and her two hypercalcemic sons.

**Conclusion:**

Familial hypocalciuric hypercalcemia due to a novel mutation in the calcium-sensing receptor gene was diagnosed in the proband and her two sons. Disorders of calcium metabolism can be multifarious in the elderly. We suggest that testing first degree relatives for calcium levels and DNA sequencing may have a role in the assessment of elderly patients with parathormone-related hypercalcemia.

## Introduction

Familial hypocalciuric hypercalcemia (FHH) is a rare autosomal dominant disease that runs a benign course. Its prevalence is not clearly established [1]. It is important to differentiate it from the much more common primary hyperparathyroidism (PHPT) to avoid unnecessary and potentially harmful parathyroidectomy [2]. It has been shown to result from heterozygous inactivating mutations in the calcium-sensing receptor (*CaSR*) gene in the majority of cases [3]. The calcium sensing receptor (CaSR) is a G-protein-coupled receptor of 1078 amino acids (AAs) with a large extracellular domain and the characteristic seven-transmembrane domains [4]. It is expressed in the parathyroid gland, kidneys, bones, and other tissues [5] and plays a key role in the maintenance of constant levels of extracellular ionized calcium. It modulates the function of chief cells of the parathyroid gland, stimulating the synthesis and secretion of PTH as well as the proliferation of parathyroid cells when the calcium level is low, and inhibiting these functions when the calcium level is high. In the kidneys, the *CaSR *decreases calcium reabsorption, increases calciuresis, and decreases the concentrating ability of the kidney when sensing hypercalcemia, through its effect on the thick ascending limb of the loop of Henle and on the medullary collecting ducts [6]. Two hundred and twenty-three mutations for the *CaSR *gene are listed in the *CaSR *mutation database [7]. Of these, 154 are inactivating (loss-of-function), and most of them cause FHH in heterozygous and neonatal severe hyperparathyroidism (NSHPT) in homozygous patients [3]. Curiously, most of these mutations are confined to a single family, with only a few having been described in more than one family. Inactivating mutations result in decreased sensing of calcium levels, shifting the calcium-PTH curve and the set-point to the right [6]. Elderly patients are frequently affected with disorders of calcium metabolism [8-10]. Here we describe an Irish family in which the proband is an octogenarian with hypercalcemia, hypocalciuria, chronic kidney disease (CKD), and low vitamin D level. Because two of her children had hypercalcemia and hypocalciuria as well, we carried out DNA sequencing in the *CaSR *gene in the patient and three of her children.

## Case presentation

An 80-year-old Irish woman was admitted to our hospital after the onset of a dense right hemiplegia and dysphasia. She had been seen in our hospital previously after an episode of collapse and was diagnosed with hypertension, atrial fibrillation, congestive heart failure, epilepsy, and hypercalcemia. She had a past history of cholecystectomy 33 years earlier. No history of constipation, anorexia, vomiting, bone pains, polyuria or polydypsia, or psychiatric or cognitive disturbance was noted. Her hypercalcemia had been quite severe, ranging between 3.22 and 3.47 mmol/L, with albumin levels of 40 g/L on both occasions; the magnesium level was 0.90 mmol/L (normal, 0.70 to 1.00). PTH levels in those instances had been measured between 170 and 235 ng/L (normal, 10 to 55 ng/L). She was taking no medication at the time of sampling. Her urine analysis was negative on three occasions. Arterial blood gases showed no evidence of an acid-base disturbance. Bicarbonate was 25 mmol/L, and chloride was 101 mmol/L. A myeloma screen was negative. Her full blood count was entirely normal. ESR was 12 mm/h. 25-OH Vitamin D level was shown to be low at 33 nmol/L (normal, 53 to 150). An X-ray showing the kidney, ureter, and bladder did not show any kidney stones or abnormal calcification.

A bone-density scan was not performed. A parathyroid sestamibi scan was normal. She was offered parathyroidectomy in another hospital; she declined. She remained at home for a period of two years without any obvious symptoms of hypercalcemia. During this admission, she was shown to have had a total left anterior circulation stroke, most probably embolic. She also had an embolism in her leg and eventually died of aspiration pneumonia.

One of her sons (son 1), reported that he was recently found to have hypercalcemia. His own past medical history included gastroesophageal reflux disease (GERD), Barrett esophagus, duodenal polyps with gastric heterotopia, asthma, allergy to shellfish, and hypercholesterolemia.

He informed us that he had seven brothers and no sister. Three of them had died: one at the age of six months of unknown cause; one at the age 26 years of an epileptic seizure; and one at the age of 36 years of pneumonia. Two were living abroad. The remaining two brothers were available for investigations. One of them (son 2), aged 37 years, was affected with hypercholesterolemia, hyperuricemia, and abnormal liver-function tests attributed to excessive alcohol intake. He also had hypercalcemia. His other brother (son 3), aged 50 years, had white-coat hypertension, hypercholesterolemia, and normocalcemia. Their results are presented in Table 1.

**Table 1:** Clinical chemistry and mutations

**Ca**^**2+**^		P	**F**^**exc **^**Ca**^**2+**^	Creatinine clearance	PTH	*CaSR *A213E mutation
2.0-2.6						

Normal values and units	mmol/L Corrected for albumin	0.8-1.5 mmol/L		ml/'	10-55 ng/L	Absent

Proband 2001	3.35	0.67		44		

Proband 2004	3.03	0.90	0.0035	48	140	Present

Son 1	2.98	0.86	0.0084	80.8	20	Present

Son 2	2.91	0.73	0.0044	97.8	68	Present

Son 3	2.40	0.93	0.0094	90.2	38	Absent

Serum calcium, phosphate, fraction of excreted calcium (calcium clearance/creatinine clearance ratio), creatinine clearance, PTH, and the presence or absence of the mutation.

**Figure 1:**
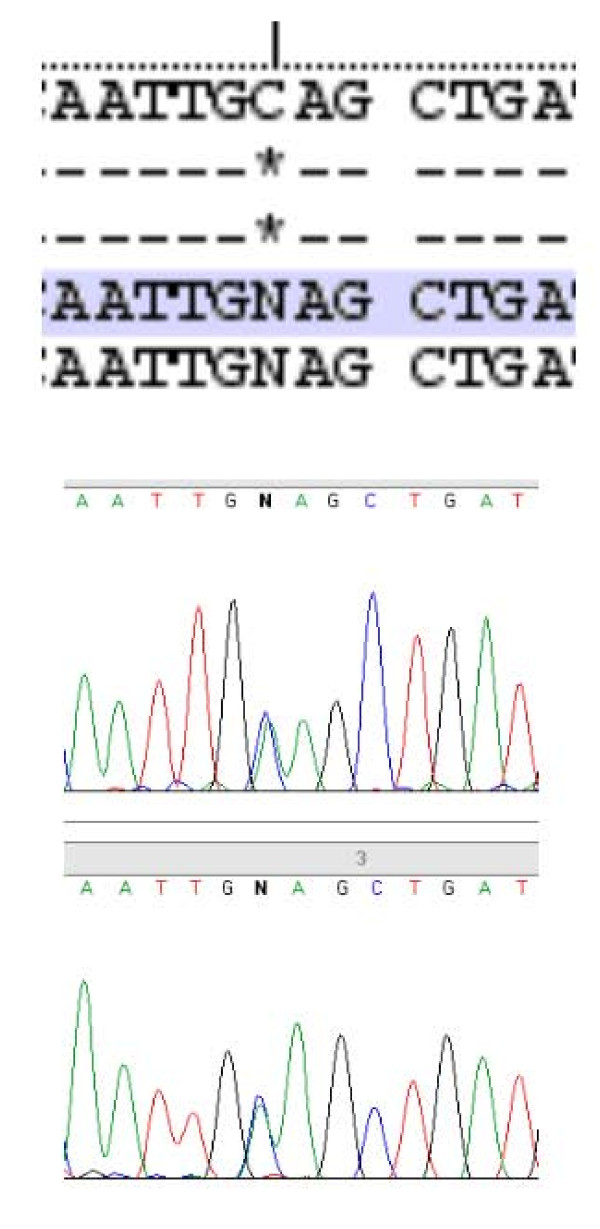
**Heterozygosity for a C > A transversion at point 213 of the *CaSR *gene of the proband**.

**Figure 2:**
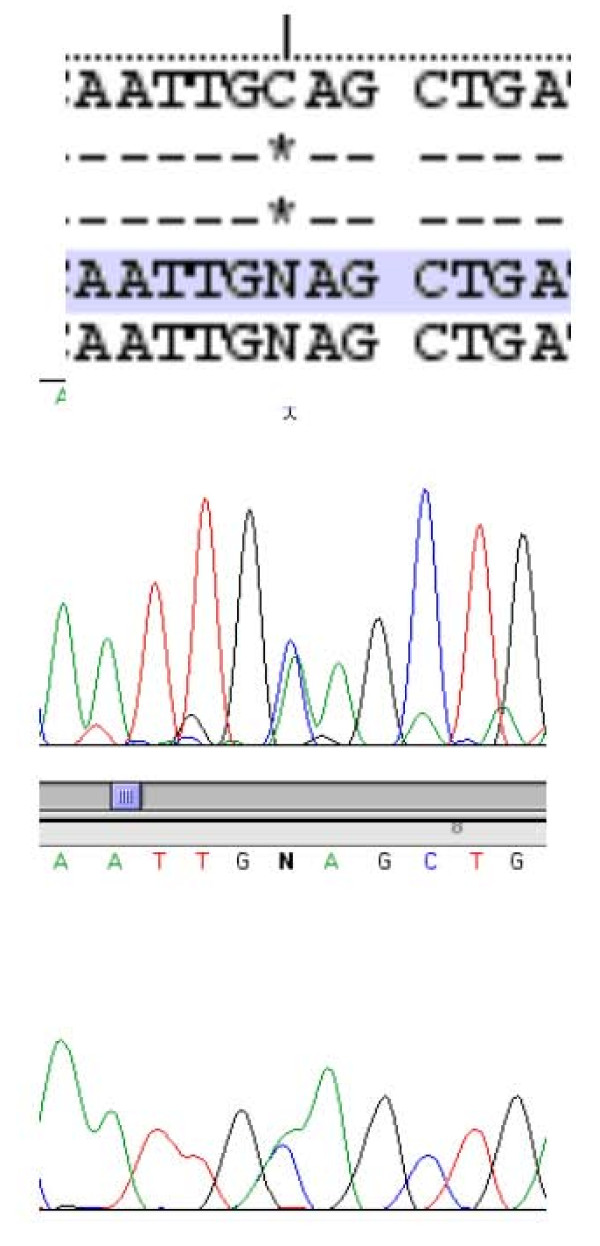
**Heterozygosity for a C > A transversion at point 213 of the *CaSR *gene in one of the affected sons of the proband**.

**Figure 3:**
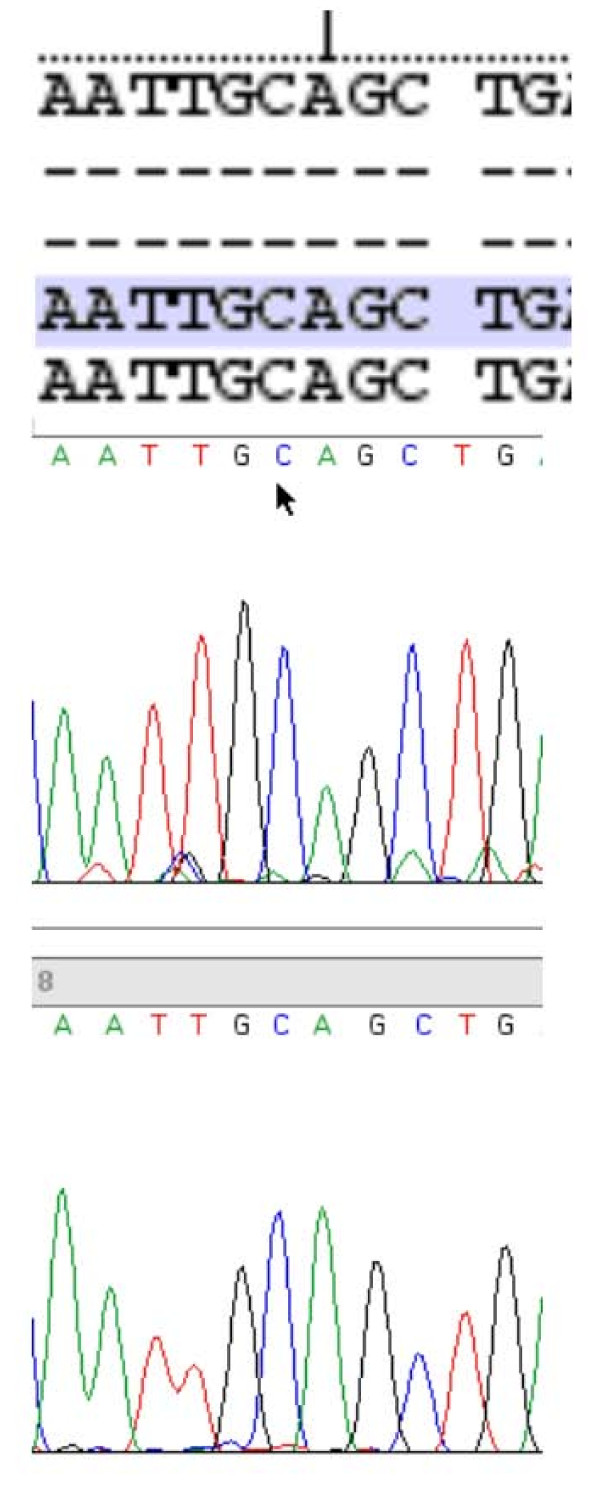
**A normal sequence of the *CaSR *gene in the unaffected son**.

The findings of a familial hypercalcemia with relative hypocalciuria were strongly suggestive of a diagnosis of familial hypocalciuric hypercalcemia (FHH). We therefore decided to analyze the calcium-sensing receptor (*CaSR*) gene. Direct DNA sequencing showed that the proband was heterozygous for a point mutation in the fourth exon of the *CaSR *gene (GCA→GAA), leading to a substitution from alanine to glutamate at position 213 (A213E) (Figure 1). Son 1 and son 2, both with hypercalcemia, were also heterozygous for the same mutation (Figure 2). Son 3, who was normocalcemic, did not carry the mutation (Figure 3). The proband, her deceased husband, and their offspring were all from the southwest of Ireland.

## Discussion

This 80-year-old woman was noted to have hypercalcemia, relative hypocalciuria, and an elevated PTH level. Possible explanations for this include the milk-alkali syndrome, the use of lithium or thiazide diuretics, primary hyperparathyroidism (PHPT) associated with vitamin D deficiency with or without low calcium intake [11], FHH, and the combination of FHH with secondary hyperparathyroidism (SHPT) and CKD. The first two possibilities are ruled out by the normal acid/base status and by the negative history of drug intake. The possibility of primary hyperparathyroidism with vitamin D deficiency exists, but the sestamibi scan was negative, and she did not appear to have any of the symptoms of hyperparathyroidism except for the hypertension and the CKD stage 3. The combination of FHH with SHPT could explain the fairly high calcium and PTH observed [12].

Two of the three sons we investigated were hypercalcemic and did carry the same GCA→ GAA mutation in the *CaSR *gene as their mother, whereas the normocalcemic son did not, strongly suggesting that the mutation was the cause of the hypercalcemia and the diagnosis of FHH. We did not test the biologic activity of this mutated receptor. This missense mutation leads to the A213E (alanine→glutamate) change in the extracellular domain of the protein, in close proximity to one of the calcium-binding sites [13]. Glutamate (acidic side chain) and alanine (nonpolar) belong to different classes of amino acids. This change is therefore likely to affect the conformation of the extracellular domain of the receptor and of its affinity for calcium. Predictive testing by using PolyPhen-2 [14] concluded that the mutation was probably damaging, with a score of 0.982 (sensitivity, 0.66; specificity, 0.94).

The phenotype of FHH in the elderly is bound to be obscured by coexisting common disorders of calcium metabolism, and conversely, the manifestations of these common disorders will be different in patients affected by FHH [8-12]. The case of our proband clearly exemplifies this. We suggest that all first-degree relatives of patients with hypercalcemia and inappropriately normal or elevated PTH levels should have a calcium level determined. We also think that DNA sequencing is minimally invasive, is becoming more affordable, can lead to accurate diagnosis, and should therefore be carried out in members of PTH-related families with hypercalcemia and hypercalcemia patients with overlapping fraction of excretion of calcium (0.01 to 0.02). This should also apply to young hypercalcemia patients, any atypical cases in which no first-degree relative is available, and those patients with a typical FHH picture with parents with normocalcemia (to detect *de novo *mutations).

Moreover, techniques such as denaturing high-performance liquid chromatography (DHPLC) seem to offer a rapid and effective way of screening for mutations in the *CaSR *gene in these patients [15]. More systematic testing would help prevent unnecessary parathyroidectomies; allow the detection of more cases, give a better idea of the prevalence of FHH and of different mutations in different populations, and help to define the phenotype, such as the set-point, associated with individual mutations.

## Conclusion

The investigation of this Irish family with hypercalcemia led to the diagnosis of FHH and to the identification of a novel mutation in the *CaSR *gene. We believe that the molecular diagnosis of FHH through DNA sequencing or DHPLC of the *CaSR *gene is clinically useful in the differential diagnosis of hypercalcemia in elderly patients with multiple comorbidities.

## Competing interests

The authors declare that they have no competing interests.

## Authors' contributions

WFE analyzed the data and prepared the manuscript. OdB managed the patients, made the diagnosis, and reviewed the manuscript. All authors read and approved the final manuscript.

## Consent

Written informed consent was obtained from the patient and the members of the family reported for publication of this case report and accompanying images. A copy of the written consent is available for review by the Editor-in-Chief of this journal.
